# Genotypic and Technological Characterization of Lactic Acid Bacteria and Coagulase-Negative Staphylococci Isolated from Sucuk: A Preliminary Screening of Potential Starter Cultures

**DOI:** 10.3390/foods14203495

**Published:** 2025-10-14

**Authors:** Mükerrem Kaya, Bilge Sayın, Kübra Çinar Topçu, Mehmet Karadayı, Aybike Kamiloğlu, Medine Güllüce, Güzin Kaban

**Affiliations:** 1 Department of Food Engineering, Faculty of Agriculture, Atatürk University, Erzurum 25240, Türkiye; gkaban@atauni.edu.tr; 2 Department of Gastronomy and Culinary Arts, School of Tourism and Hotel Management, Ardahan University, Ardahan 75002, Türkiye; bilgesayin@ardahan.edu.tr; 3 Aydıntepe Vocational School, Department of Food Processing, Bayburt University, Bayburt 69500, Türkiye; cinar.kbr@gmail.com; 4 Department of Biology, Faculty of Science, Atatürk University, Erzurum 25240, Türkiye; mkaradayi@atauni.edu.tr (M.K.); gullucem@atauni.edu.tr (M.G.); 5 Department of Food Engineering, Faculty of Engineering, Bayburt University, Bayburt 69000, Türkiye; abereketoglu@bayburt.edu.tr

**Keywords:** lactic acid bacteria, coagulase-negative staphylococci, sucuk, dry fermented sausage, genetic identification, antagonistic activity, antibiotic resistance

## Abstract

This study aimed to characterize lactic acid bacteria (LAB) and coagulase-negative staphylococci (CoNS) isolated from traditionally produced sucuk for their potential use in starter culture development and food safety applications in fermented meat products. A total of 145 isolates (95 LAB and 50 CoNS) were analyzed through genetic identification, phylogenetic analysis, and assessments of technological properties. Antagonistic activity against *Listeria monocytogenes* and *Staphylococcus aureus* was also evaluated, along with antibiotic sensitivity. Among LAB, *Lactiplantibacillus plantarum* was the most prevalent species (60 isolates), while *Staphylococcus xylosus* was the predominant CoNS species (24 isolates). The isolates exhibited diverse technological properties and varying levels of antagonistic activity against the tested pathogens. Antibiotic sensitivity tests indicated that 15 selected isolates were negative for antibiotic resistance genes. Overall, this comprehensive characterization provides valuable insights for the development of starter cultures and for enhancing food safety in fermented meat products.

## 1. Introduction

Fermented meat products are foods that acquire their desired properties through the activity of microorganisms and/or enzymes. Fermented sausages represent a significant proportion of these products and are widely consumed across many countries. In Türkiye, two types of fermented sausages are produced: sucuk and heat-treated sucuk. Sucuk refers to dry-fermented sausage produced without a subsequent heat treatment step, while heat-treated sucuk denotes a semi-dry fermented sausage produced by short fermentation followed by heat treatment (60–68 °C) and drying. In both products, starter cultures are crucial for acid formation during the fermentation phase and, therefore, especially for ensuring product safety. However, while starter culture activity continues throughout the ripening process in sucuk, the activity is limited in heat-treated sucuk due to the heat treatment [1]. The use of starter cultures has been increasing for both product types. However, industrially produced sucuk often falls short of consumer expectations, particularly in terms of taste and aroma. Therefore, the selection of appropriate starter culture preparations is crucial [2]. Since the dominant microbiota of traditional fermented sausages is associated with desirable characteristics, using selected strains as starter cultures can contribute to preserving the typical character of the product, as well as providing benefits for both producers and consumers [3,4]. The most suitable approach is to isolate strains with desirable technological characteristics from fermented sausages and then apply them as starter cultures [5].

Two groups of microorganisms are considered technologically important in the fermentation and ripening of fermented meat products. Lactic acid bacteria (LAB) contribute not only to flavor development, but also to the inhibition of spoilage organisms and foodborne pathogens such as *Listeria monocytogenes* and *Staphylococcus aureus* [2,6]. Gram-positive, catalase-positive cocci (mainly coagulase-negative staphylococci) represent the other important group, contributing to aroma formation through their proteolytic and lipolytic activities, preventing or delaying autoxidation by decomposing peroxides through catalase activity, and playing a role in color formation via nitrate reductase activity [2,7,8].

Commercial starter culture preparations for meat products were first introduced in the USA in 1957 and in Europe in 1961. These preparations are available as single- or multi-strain cultures and are usually derived from traditional products. In Türkiye, a limited number of studies have focused on the isolation and identification of LAB and coagulase-negative staphylococci (CoNS) from sucuk [9,10,11,12]. Moreover, only three studies have tested strains isolated from sucuk as starter cultures [13,14,15]. Thus, there is a clear need for more comprehensive research on the development of starter cultures in meat products. Starter cultures are used in the meat industry to ensure product safety by inhibiting pathogenic bacteria, to prolong shelf life by preventing undesirable changes caused by spoilage microorganisms or abiotic reactions, to provide novel sensory properties, and to confer beneficial health effects [2].

Among LAB, strains of *Lactiplantibacillus plantarum*, *Latilactobacillus curvatus*, *Lactiplantibacillus pentosus*, *Latilactobacillus sakei*, *Pediococcus pentosaceus*, and *P. acidilactici* are widely used as starter cultures in the meat industry. Among Gram-positive, catalase-positive cocci, *Staphylococcus carnosus*, *Staphylococcus xylosus*, and *Kocuria varians* are common [2,12,13]. In the present study, LAB and CoNS isolates obtained from traditionally produced sucuk were genotypically identified, and their technological properties as well as antibiotic sensitivities were evaluated. The potential for utilizing these strains as starter cultures was then evaluated.

## 2. Materials and Methods

### 2.1. Material

In this study, molecular characterization was performed on 95 isolates of lactic acid bacteria (LAB) and 50 isolates of coagulase-negative staphylococci (CoNS) previously isolated and identified by Kaban [16] and stored at −80 °C. Molecular identification was carried out based on the homology of the 16S rRNA region. To determine the technological properties of LAB isolates tests were conducted for growth at different temperatures, NaCl and pH tolerance, lipolytic and proteolytic activities, acetoin formation, D-/L-lactic acid production, and amino acid decarboxylase activity. In addition, agar spot and well diffusion assays were performed to evaluate antagonistic activity. For CoNS isolates, growth at different temperatures, NaCl concentrations, and pH values, as well as proteolytic and lipolytic activities, acetoin production, biofilm formation, and nitrate reductase activity were assessed. Finally, the antibiotic sensitivities of all isolates were determined.

### 2.2. DNA Isolation and 16s rRNA Amplification

Genomic DNA was isolated according to the method described by Barış [17]. For the identification of bacterial isolates, the 16S rRNA gene region was selected and amplified in vitro using universal primers 27F (forward 5′-AGA GTT TGA TCC TGG CTC AG-3′; 0.7 µL, 50 µM) and 1492R (reverse 5′-GGT TAC CTT GTT ACG ACT T-3′; 0.7 µL, 50 µM) in a 70 µL polymerase chain reaction (PCR). The PCR mixture contained 7 µL of 10× PCR buffer (100 mM Tris–HCl, 500 mM KCl, 15 mM MgCl_2_, 0.01% gelatin, pH 8.3), 1.4 µL of dNTP mix (10 mM each of dATP, dGTP, dCTP, dTTP), 2.8 µL of DMSO, 4.2 µL of MgCl_2_, 0.7 µL of Taq DNA polymerase (5 U/µL), 1.5 µL of template DNA, and sterile distilled water to adjust the final volume.

The PCR conditions were as follows: initial denaturation at 95 °C for 2 min, followed by 36 cycles of denaturation at 94 °C for 1 min, annealing at 53 °C for 1 min, and extension at 72 °C for 2 min, with a final extension step at 72 °C for 5 min [14]. PCR products were analyzed by electrophoresis in 1% agarose gel containing 0.6 µL ethidium bromide, run at 90 V for 75 min, and visualized using a gel documentation system (DNR BioImaging Systems Software version 2.7). Sequencing of the PCR products was performed by MacroGen Inc. (Seoul, Republic of Korea). The resulting sequences were deposited in the NCBI database, and accession numbers were obtained for the identified isolates. Multiple sequence alignment of the 16S rRNA gene sequences was conducted using MEGA X 10.1.7, and a phylogenetic tree was constructed by the neighbor-joining (NJ) method with 1000 bootstrap replicates [18]. MEGA X software was also used for further phylogenetic analyses [19]. It should be noted that 16S rRNA sequence analysis, while providing reliable identification at the genus and species levels, has inherent limitations in distinguishing strain-level differences due to the highly conserved nature of this genetic region. The molecular identification performed in this study should therefore be considered as species-level identification rather than strain-level characterization.

### 2.3. Determination of Technological Properties

#### 2.3.1. Growth at Different Temperatures

The growth ability of LAB and CoNS isolates was evaluated at 4 °C (7–10 days), 15 °C (3 days), 25 °C (2 days), and 45 °C (3 days). Isolates were incubated in de Man, Rogosa and Sharpe (MRS, Merck, Darmstadt, Germany) broth (for LAB) and Brain Heart Infusion (BHI, Merck, Darmstadt, Germany) broth (for CoNS). Optical density at 600 nm was measured using a spectrophotometer to assess growth [20].

#### 2.3.2. Growth at Different NaCl Concentrations

To determine salt tolerance, LAB and CoNS isolates were incubated in MRS and BHI broth supplemented with 6.5% and 10% NaCl (*w*/*v*) at 30 °C for 5 days. Growth was monitored by measuring optical density at 600 nm [20].

#### 2.3.3. Growth at Different pH Values

Overnight cultures (0.05 mL) of LAB isolates were inoculated into MRS broth, and CoNS isolates into BHI broth, both adjusted to different pH values using HCl. After incubation at 32 °C for 18–24 h, the presence of turbidity was recorded as positive [21].

#### 2.3.4. Lipolytic Activity

Supernatants of LAB and CoNS isolates were inoculated into wells of tributyrin agar (Merck, Darmstadt, Germany) and incubated at 30 °C for 6 days. Lipolytic activity was determined by the appearance of clear zones around the wells [22].

#### 2.3.5. Proteolytic Activity

Proteolytic activity was determined using gelatinase and calcium caseinate agar (Merck, Darmstadt, Germany). LAB and CoNS trains were inoculated onto the media, and the presence of clear zones around colonies was considered positive [23].

#### 2.3.6. Acetoin Formation

Acetoin production from glucose was tested using Methyl Red/Voges-Proskauer (MR/VP) medium (Merck, Darmstadt, Germany). Tubes containing 5 mL of medium were sterilized at 121 °C for 15 min and inoculated with overnight cultures. After incubation at 32 °C for 48 h, 1 mL of culture was mixed with 0.2 mL of 40% KOH and 0.6 mL of α-naphthol solution. The appearance of a pink to bright red ring within 15 min indicated a positive result [24].

#### 2.3.7. Determination of Antibiotic Sensitivities

Antibiotic sensitivity of LAB and CoNS isolates was determined using the disk diffusion method. The following antibiotic disks were used: ampicillin (10 µg), clindamycin (2 µg), erythromycin (15 µg), gentamicin (10 µg), kanamycin (30 µg), tetracycline (30 µg), vancomycin (30 µg), streptomycin (10 µg), cephalothin (30 µg), and penicillin G (10 U). Disks were placed on Mueller–Hinton agar (Merck, Darmstadt, Germany) plates, and zones of inhibition were measured after incubation [23].

#### 2.3.8. D-L Lactic Acid Production

D-/L-lactic acid production by LAB isolates was determined using enzyme test kits (Boehringer Mannheim R–Biopharm AG, Darmstadt, Germany) according to the manufacturer’s instructions [25].

#### 2.3.9. Amino Acid Decarboxylase Activity

Møller decarboxylase medium (Merck, Darmstadt, Germany) was used to assess biogenic amine formation in LAB isolates. Four media were prepared: one control (no amino acid added) and three supplemented with arginine, lysine, or ornithine (5 g/L each). Each tube was filled with 3 mL of medium and overlaid with 5 mm of sterile paraffin oil. After sterilization at 115 °C for 10 min, isolates were inoculated and incubated at 25 °C for 7 days. A color change from yellow to purple indicated positive decarboxylase activity [24].

#### 2.3.10. Antagonistic Activity

Antagonistic activity of LAB isolates against *Listeria monocytogenes* (ATCC 7644) and *Staphylococcus aureus* (ATCC 25923 and ATCC 29213) was assessed by agar spot and well diffusion assays [26].

Agar spot test: LAB isolates were spotted on MRS plates and incubated anaerobically overnight. Plates were overlaid with semi-solid tryptic soy agar (TSA, 0.8% agar) containing 100 µL of pathogen culture, then incubated aerobically at 37 °C for 48 h. Zone diameters were measured.

Well diffusion test: Cell-free supernatants from overnight cultures were obtained by centrifugation (25 °C, 5700 g, 10 min), neutralization to pH 6.5–7.0 and filtration (cellulose acetate filter). Wells (5 mm) were made in TSA plates containing 100 µL of pathogen culture. Each well was filled with 50 µL of supernatant and plates were incubated aerobically at 37 °C for 48 h. The antimicrobial activity was assessed by measuring inhibition zones.

#### 2.3.11. Nitrate Reductase Activity

CoNS isolates were inoculated into nitrate broth (Merck, Darmstadt, Germany) and incubated at 35 °C for 24 h. Nitrate reagent (0.2–0.5 mL) was then added. The appearance of a red color indicated a positive result. If no color appeared, zinc dust was added for confirmation: red color after zinc addition was considered negative [24].

#### 2.3.12. Biofilm Formation

Biofilm formation by CoNS isolates was determined on Congo red agar (CRA, Merck, Darmstadt, Germany) according to the method of Landeta et al. [23]. CRA plates were prepared with 0.8 g Congo red, 0.8 g sucrose, 37 g MRS broth, and 10 g agar in 1 L of medium. Plates were incubated at 37 °C for 24 h and then at room temperature overnight. Biofilm-producing isolates formed black colonies, while non-producers formed red colonies.

### 2.4. Identification of Antibiotic Resistance Genes

Fifteen strains of LAB and CoNS were selected from the molecularly identified and technologically characterized isolates (*Latilactobacillus sakei* S15, *L. sakei* S23, *L. plantarum* S24, *L. plantarum* S33, *L. plantarum* S40, *L. plantarum* S91, *L. plantarum* S93, *L. plantarum* S107, *Pediococcus pentosaceus* S128b, *P. acidilactici* S145a, *Staphylococcus xylosus* G27, *S. saprophyticus* G13, *S. xylosus* S98, *S. xylosus* S81, and *S. carnosus* G109), and analyzed for antibiotic resistance genes against penicillin G, ampicillin, clindamycin, erythromycin, gentamicin, kanamycin, tetracycline, streptomycin, cephalotin, and vancomycin. To confirm phenotypic resistance, the corresponding resistance genes were screened genotypically. PCR assays were performed using specific primers for the antibiotic resistance genes listed in Table 1, and the amplification conditions were directly adopted from the referenced studies from which the primers were obtained. The resulting PCR products were analyzed by electrophoresis in 1.5% agarose gel prepared with 0.5× TBE buffer and containing ethidium bromide.

## 3. Results and Discussion

### 3.1. Molecular Identification

In this study, 95 lactic acid bacteria (LAB) isolates and 50 coagulase-negative staphylococci (CoNS) isolates were identified by 16S rRNA analysis. LAB accession numbers included KR025381–KR025399, KX831552–KX831555, KR011002–KR011009, KR011012–KR011016, KR011018–KR011022, KR010996–KR010999, KT327837–KT327866, KR025400–KR025405, and KT275940–KT275959. For catalase-positive cocci, the accession numbers were KT3728370119–KT3728370125.

Genetic identification revealed that 60 isolates were identified as *Lactiplantibacillus* (formerly *Lactobacillus*) *plantarum*, followed by 18 isolates of *L. paraplantarum*, 13 isolates of *Latilactobacillus* (formerly *Lactobacillus*) *sakei*, three isolates of *Pediococcus acidilactici*, and one isolate of *P. pentosaceus*. Thus, the dominant species in sucuk samples was *L. plantarum*, consistent with the findings of Kaban [16]. However, *L. sakei* and *L. paraplantarum* were also identified through genotypic analysis. Similarly, 37.2% [20] and even 45.6% [21] of isolates from Greek-type traditional fermented sausages were reported as *L. plantarum*. In Spanish-type sausages, *L. plantarum* was detected in 50% of fuet samples and in 100% of chorizo samples [34]. Drosinos et al. [20] further identified 7.3% of LAB isolates as *L. curvatus* and 3.5% as *L. sakei*. Although not dominant, *L. sakei* was also isolated in sucuk studies by Gürakan et al. [9] and Çon and Gökalp [11], whereas Özdemir [10] reported *L. sakei* as the dominant species. Adigüzel and Atasever [35] identified *L. plantarum* as dominant in sucuk by both phenotypic and genotypic methods, while also isolating *P. pentosaceus*, *Lactococcus lactis* subsp. *lactis*, *L. curvatus* subsp. *curvatus*, *L. brevis*, *L. fermentum*, *Weissella viridescens*, *L. delbrueckii* subsp. *delbrueckii*, *W. confusa*, *L. collinoides*, and *Leuconostoc mesenteroides* subsp. *mesenteroides* var. *dextranicum*. These variations are attributed to the absence of standardized sausage production methods and differences in ripening temperature as well as the type and dosage of curing agents. Kaya and Kaban [2] noted that *L. curvatus* and *L. sakei* were dominant in traditional fermented sausages fermented at 20–22 °C without starter culture, whereas *L. plantarum* prevailed at higher ripening temperatures (>25 °C). In other European countries, *L. sakei*, *L. curvatus*, and *L. plantarum* have been reported as dominant species in traditional fermented sausages [36].

In addition to lactobacilli species, three isolates were identified as *P. acidilactici* and one as *P. pentosaceus*. These species were also isolated by Çon and Gökalp [11], while Yaman et al. [37] reported only *P. pentosaceus*. Different proportions of *Pediococcus* species have been reported in other types of fermented sausages [38,39].

Among CoNS isolates, *Staphylococcus xylosus* was identified as the dominant species (24 isolates), followed by *S. saprophyticus* (10 isolates). Other identified species included *S. equorum* (6 isolates), *S. simulans* (3 isolates), *S. succinus* (2 isolates), *S. carnosus* (2 isolates), *S. hominis* (1 isolate), *S. caprae* (1 isolate), and *S. vitulinus* (1 isolate). These findings were consistent with the phenotypic description by Kaban [16]. A notable finding was the dominance of *S. xylosus*. Among catalase-positive cocci, *Staphylococcus* species are generally more prevalent than micrococci in dry fermented sausages [40]. *S. xylosus* has been reported as the dominant species in many Italian [41,42,43,44] and Spanish-type fermented sausages [45,46,47]. In contrast, *S. saprophyticus* has been identified as dominant in some other fermented sausages [20,21,48,49,50].

*S. carnosus*, a species commonly used as a starter culture, was also detected, though its isolation rate was low [16]. In fact, *S. carnosus* has not been recovered in several studies [42,44,46,47,48,50]. Nevertheless, Montel et al. [51], Papamanoli et al. [49], Aymerich et al. [34], and Martin et al. [47] succeeded in isolating *S. carnosus* from fermented sausage samples.

Another technologically important species is *S. equorum*. Due to its salt tolerance, ability to grow at low temperatures, and proteolytic and lipolytic activities, *S. equorum* has been suggested as a potential starter culture for fermented meat products [52]. In this study, six isolates were identified as *S. equorum*, and this species has also been reported in other fermented sausages [21,42,44,50,53]. By contrast, *S. carnosus* was isolated only rarely; for example, Nunes et al. [54] detected *S. carnosus* in commercial salami but not in artisanal products.

**Figure 1:**
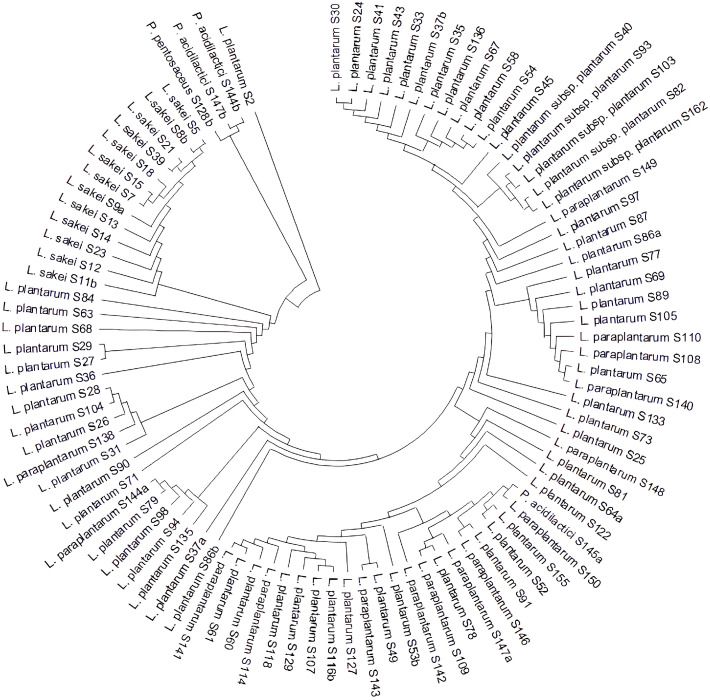
The phylogenetic tree constructed from the 16S rRNA regions of LAB (The phylogenetic tree of all LAB isolates (*n* = 95) was constructed based on 16S rRNA gene sequences. The Neighbor-Joining method was used to generate the tree [18], and the optimal tree had a total branch length of 0.21319605. Evolutionary distances were calculated using the p-distance method [55]. The final dataset included 1464 positions, and evolutionary analyses were performed using MEGA X [19]).

According to the phylogenetic tree shown in Figure 1, most *L. plantarum* strains and all *L. paraplantarum* strains clustered closely together, whereas *L. plantarum* S2 formed a separate subgroup. Thirteen *L. sakei* strains grouped closely into a distinct cluster, while the three *Pediococcus* strains also formed a subgroup. Interestingly, the *P. acidilactici* S145a was included in a subgroup with *L. plantarum* and *L. paraplantarum* (Figure 1). A close phylogenetic relationship between *Pediococcus* and *Lactobacillus* species has also been reported previously [55]. Evaluation of the growth characteristics of the *P. acidilactici* S145a revealed that, unlike the other *P. acidilactici* strains, it exhibited good growth at pH 4.5 (Figure 1).

**Figure 2:**
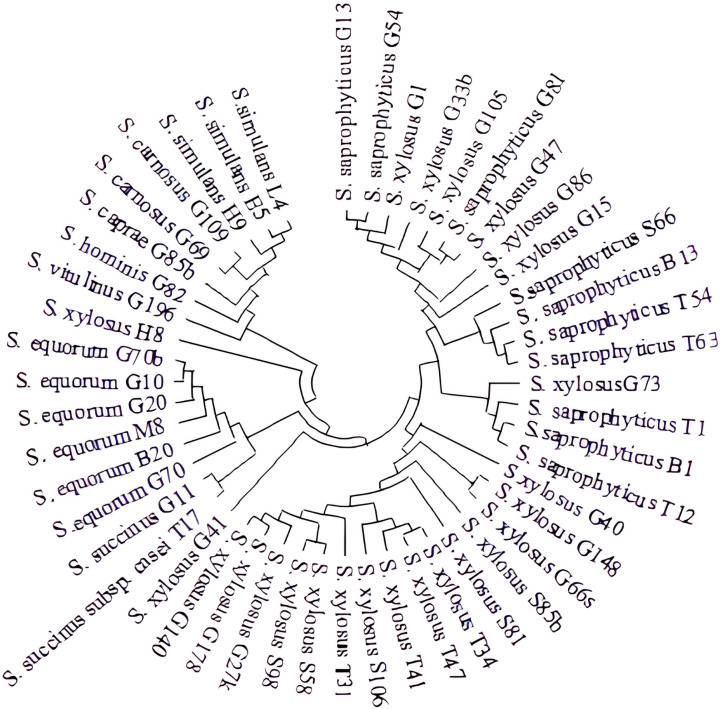
The phylogenetic tree constructed from the 16S rRNA regions of *Staphylococcus* strains (The Neighbor-Joining method [18] was used to infer the evolutionary history. The optimal tree is presented, with a total branch length of 0.14661326. Related taxa clustered together according to the bootstrap test (1000 replicates) [56]. Evolutionary distances were calculated using the p-distance method [53]. A total of 50 CoNS nucleotide sequences were analyzed, and the final dataset contained 1485 positions. Evolutionary analyses were performed using MEGA X [16]).

The phylogenetic tree constructed from the 16S rRNA regions of *Staphylococcus* strains is presented in Figure 2. They formed seven subgroups. One of these subgroups consisted exclusively of *S. xylosus* strains, which clustered closely with *S. saprophyticus*. In other subgroups, *S. equorum* and *S. succinus* grouped together, while *S. vitulinus, S. hominis*, *S. caprae*, *S. simulans*, and *S. carnosus* were found to be phylogenetically close (Figure 2). A similar affinity between these strains was also reported by Nunes et al. [54].

### 3.2. Technological Properties

Growth characteristics of the isolates are shown in Table 2. The ability of LAB isolates to grow under different temperature conditions is a key factor in determining their suitability as starter cultures, since it directly influences fermentation performance and the rate of acid formation. Strain characteristics also affect the textural and sensory properties of fermented products. Environmental factors such as fermentation temperature, pH, and salt concentration in sucuk and similar dry fermented sausages are critical for the survival and activity of starter cultures. Importantly, isolates to be used as starters should not exhibit amino acid decarboxylase activity, as this leads to the formation of biogenic amines. For CoNS, catalase and nitrate reductase activities are particularly important for color formation, oxidative stability, and overall product quality. In addition, proteolytic and lipolytic activities in this group of microorganisms contribute significantly to flavor development [57].

All 60 *L. plantarum* isolates exhibited strong growth at 25 °C, and all but one isolate (*L. plantarum S53b*) grew well at 15 °C. Drosinos et al. [20] reported that 86% of *L. plantarum* isolates grew at 4 °C, 100% at 15 °C, and 32.7% at 47 °C. In contrast, CoNS isolates generally did not grow at 4 °C, and no isolates grew well at 45 °C. As shown in Table 2, the majority of LAB grew very well at 6.5% NaCl, whereas growth decreased at 10% NaCl, with only three isolates showing good tolerance. Similarly, a considerable number of CoNS isolates grew at 6.5% NaCl. pH also proved to be an important factor, as most isolates grew well between pH 5.0 and 6.5. The acidification that occurs during fermentation, and the resulting drop in pH, not only influences color, flavor, and texture development but also inhibits spoilage and pathogenic microorganisms [2]. However, a pH drop below 5 can negatively impact flavor formation due to the suppression of catalase-positive cocci [16].

Biochemical and metabolic activities of LAB and CoNS isolates were given in Table 3. Most of the tested isolates did not show proteolytic or lipolytic activity. However, seven *L. plantarum* isolates and one *L. sakei* strain exhibited strong proteolytic activity. Among CoNS, 42% of *S. xylosus* isolates showed proteolytic activity. With respect to lactic acid configuration, all but three *L. plantarum* isolates produced D-L lactic acid, as did 11 *L. sakei* isolates and all *Pediococcus* isolates. Amino acid decarboxylase activity was rare: only one strain (*L. plantarum* S97, with arginine decarboxylase activity) tested positive.

One of the most important functions of CoNS in meat products is their nitrate reductase activity. This activity is particularly significant in products containing nitrate, as microorganisms capable of converting nitrate to nitrite are required for the expected effects of nitrate to occur [58]. In the present study, nitrate reductase activity was observed in all isolates except six CoNS isolates (*S. xylosus* [*n* = 5] and *S. carnosus* [*n* = 1]). LAB isolates may also display nitrate reductase activity. Kamiloğlu et al. [59] reported weak nitrate reductase activity in *L. plantarum* isolates from sucuk. In the present study, seven *L. plantarum* isolates, one *L. sakei* strain, and one *L. paraplantarum* strain showed nitrate reductase activity. Additionally, 48 CoNS isolates (except two, *S. xylosus* and *S. equorum*) produced acetoin. Likewise, 86% of LAB isolates were acetoin-positive. Excessive acetoin or acetic acid formation in fermented sausages is considered undesirable [2].

Biofilm formation was also evaluated in CoNS isolates, with only one isolate (*S. equorum*) testing positive. In this study, the Congo Red Agar (CRA) method was employed as a screening tool to assess biofilm formation. While CRA is a simple and cost-effective method, it has recognized limitations in sensitivity and accuracy. Nevertheless, it provided valuable preliminary data that can guide future, more quantitative investigations. Biofilm formation is an important virulence factor and may contribute to the pathogenic potential of microorganisms in humans [60].

Finally, the antimicrobial activities of LAB highlight their potential as natural barriers for product safety. LAB can exert antimicrobial effects through the production of organic acids (lactic, acetic, formic, propionic), proteinaceous bacteriocins, hydrogen peroxide, and other inhibitory substances [61]. These properties enhance both the safety and technological performance of starter cultures in fermented meat products.

Agar spot and well diffusion assays were performed to evaluate the antagonistic activity of the isolates identified in this study. In the agar spot test, all isolates exhibited good to very good antagonistic activity against *Listeria monocytogenes*, whereas fewer isolates were active against *Staphylococcus aureus*. In contrast, in the well diffusion assay, only one *L. plantarum* strain demonstrated antagonistic activity against *L. monocytogenes* (Table 4). The discrepancy between agar spot and well diffusion results may be explained by the nature of inhibitory compounds. In agar spot assays, bacterial colonies produce high local concentrations of acids, hydrogen peroxide, or bacteriocins directly on the agar surface, resulting in visible inhibition. In contrast, cell-free supernatants tested in well diffusion may contain lower concentrations of inhibitory molecules, some of which may not diffuse efficiently in agar. Moreover, inhibition observed without pH neutralization or catalase treatment may reflect organic acids or hydrogen peroxide rather than bacteriocins [6].

Antibiotic sensitivity and the absence of antibiotic resistance genes are also critical criteria for selecting isolates to be used as starter cultures. According to the World Health Organization, LAB intended for food applications should not harbor resistance to clinically relevant antibiotics [62]. Antibiotic susceptibility of the identified isolates was assessed against 10 antibiotics (Table 5). As shown, most LAB isolates were susceptible to ampicillin, clindamycin, erythromycin, and tetracycline, but resistant to vancomycin, streptomycin, kanamycin, and gentamicin. In contrast, the majority of CoNS isolates were sensitive to all antibiotics tested. Wang et al. [63] examined the antibiotic susceptibility and resistance genes of LAB and staphylococci isolates obtained from Chinese cured beef samples. They reported ampicillin sensitivity in all isolates, as well as sensitivity to penicillin, gentamicin, neomycin, norfloxacin, and ciprofloxacin at low concentrations. However, high levels of streptomycin resistance were observed, and widespread resistance was reported for vancomycin, erythromycin, roxithromycin, lincomycin, and kanamycin. The effects of tetracycline, oxytetracycline, and chloramphenicol varied among isolates.

In the present study, the genomic DNA of selected isolates intended for use in sausage production was screened for resistance genes against penicillin G, ampicillin, clindamycin, erythromycin, gentamicin, kanamycin, tetracycline, streptomycin, cephalothin, and vancomycin. No antibiotic resistance genes corresponding to the tested antibiotics were detected in any of the isolates.

## 4. Conclusions

This study provided a detailed characterization of lactic acid bacteria (LAB) and coagulase-negative staphylococci (CoNS) obtained from traditionally fermented sucuk. Among LAB, *Lactiplantibacillus plantarum* was the most frequent species, while *Staphylococcus xylosus* dominated within the CoNS group. The isolates evaluated for their potential as starter cultures demonstrated good growth in both heat-treated sucuk and traditionally sucuk. In addition, they exhibited key technological properties—including nitrate reductase activity, antagonistic activity, D-/L-lactic acid production, and proteolytic and lipolytic activities—indicating their suitability for use as starter cultures. The rate and extent of acid formation are critical not only for product characteristics but also for ensuring microbiological stability and safety in sucuk production. In this traditional fermented meat product, a continuous and technologically relevant decrease in pH during fermentation can only be achieved through the use of starter cultures. Moreover, the application of starter cultures allows for the production of standardized and high-quality products. In conclusion, the use of genotypically characterized LAB isolated from traditionally produced sucuk, together with CoNS isolates selected for desirable technological traits, may provide significant contributions to both the quality and safety of sucuk and heat-treated sucuk.

## Figures and Tables

**Table 1:** Primer sets used to identify antibiotic resistance genes.

Antibiotic		Primers	References
Penicillin G	blaZ	5′-ACTTCAACACCTGCTG TTTC-3′/5′-TGACCACTTTTATCAGCAACC-3′	Martineau et al. [27]
Ampicillin	blaTEM	5′-GCGGAACCCCTATTTG-3′/5′-ACC AAT GCT TAA TCA GTG AG-3′	Maynou et al. [28]
Clindamycin	linB	5′-CCTACCTATTGTTTGTGGAA-3′/5′-ATAACGTTACTCTCCTATTC-3′	Bozdogan et al. [29]
Erythromycin	erm (A)	5′-AAGCGGTAAAACCCCTCTGAG-3′/5′-TCA AAG CCT GTC GGA ATT GG-3′	Ouoba et al. [30]
Gentamicin	aac(3″)IV	5′-AGTTGACCCAGGGCTGTCGC-3′/5′-GTG TGC TGC TGG TCC ACA GC-3′	Ouoba et al. [30]
Kanamycin	aph(3″)-I	5′-AACGTCTTGCTCGAGGCCGCG-3′/5′-GGCAAGATCCTGGTATCGGTCTGCG-3′	Ouoba et al. [30]
Tetracycline	tetA	5′-GTAATTCTGAGCACTGTCGC-3′/5′-CTGCCTGGACAACATTGCTT-3′	Sáenz et al. [31]
Streptomycin	strA	5′-CCAATCGCAGATAGAAGG C-3′/5′-CTT GGT GAT AAC GGC AAT TC-3′	Ouoba et al. [30]
Cephalotin	blaCTX-M9	5′-GTGACAAAGAGAGTGCAACGG-3′/5′-ATGATTCTCGCCGCTGAAGCC-3′	Jafari et al. [32]
Vancomycin	vanR	5′-AGCGATAAAATACTTATTGTGGA-3′/5′-CGGATTATCAATGGTGTCGTT-3′	Dezfulian et al. [33]

**Table 2:** Growth characteristics of isolates at different temperature, pH and NaCl concentrations.

		Isolates													
		L1	L2	L3	P1	P2	S1	S2	S3	S4	S5	S6	S7	S8	S9
		Number of Isolates													
		60	18	13	3	1	24	10	6	3	2	2	1	1	1
4 °C	N	-	-	-	-	-	21	9	4	3	1	2	-	1	1
	P	60	18	13	3	1	3	1	2	-	1	-	1	-	-
15 °C	N	-	-	-	-	-	1	-	-	-	-	-	-	-	-
	P	-	-	-	-	-	3	1	3	3	-	1	-	-	-
	G	1	2	13	1	-	20	9	3	-	1	1	1	1	1
	VG	59	16	-	2	1	-	-	-	-	1	-	-	-	-
25 °C	P	-	-	-	-	-	2	-	2	-	-	-	-	-	-
	G	-	-	4	1	-	22	10	4	3	2	2	1	1	1
	VG	60	18	9	2	1	-	-	-	-	-	-	-	-	-
45 °C	N	1	-	-	-	-	13	7	4	-	1	1	-	1	1
	P	7	-	11	-	-	11	3	2	3	1	1	1	-	-
	G	52	18	2	3	1	-	-	-	-	-	-	-	-	-
6.5% NaCl	N	2	-	-	-	-	-	-	-	-	-	-	-	-	-
	P	-	-	8	-	-	4	1	-	-	-	-	-	-	-
	G	-	-	4	2	-	20	9	6	3	2	2	1	1	1
	VG	58	18	1	1	1	-	-	-	-	-	-	-	-	-
10% NaCl	N	10	1	13	-	-	1	-	-	-	-	-	-	-	-
	P	47	17	-	3	1	4	1	-	1	-	1	-	-	1
	G	3	-	-	-	-	19	9	6	2	2	1	1	1	-
pH 4.5	N	-	-	7	-	-	6	1	2	-	-	-	-	-	-
	P	3	-	2	2	-	14	3	3	3	2	2	1	1	-
	G	57	18	4	1	1	4	6	1	-	-	-	-	-	1
pH 5.0	N	-	-	-	-	-	3	-	2	-	-	-	-	-	-
	P	2	-	8	-	-	7	3	2	-	1	1	1	1	-
	G	57	18	5	3	1	14	7	2	3	1	1	-	-	1
	VG	1	-	-	-	-	-	-	-	-	-	-	-	-	-
pH 5.5	P	1	-	-	-	-	5	-	3	-	-	1	-	-	-
	G	-	-	10	2	-	19	10	3	3	2	1	1	1	1
	VG	59	18	3	1	1	-	-	-	-	-	-	-	-	-
pH 6.0	P	-	-	-	-	-	4	-	2	-	-	-	-	-	-
	G	-	-	8	2	-	20	10	4	3	2	2	1	1	1
	VG	60	18	5	1	1	-	-	-	-	-	-	-	-	-
pH 6.5	P	-	-	-	-	-	2	-	2	-	-	-	-	-	-
	G	-	-	8	2	-	22	10	4	3	2	2	1	1	1
	VG	60	18	5	1	1	-	-	-	-	-	-	-	-	-

L1: *L. plantarum*; L2: *L. paraplantarum*; L3: *L. sakei*; P1: *P. acidilactici*; P2: *P. pentosaceus*; S1: *S. xylosus*; S2: *S. saprophyticus*; S3: *S. equorum*; S4: *S. simulans*; S5: *S. succinus*; S6: *S. carnosus*; S7: *S. hominis*; S8: *S. caprea*; S9: *S. vitulinus*, N: negative, P: poor, G: good, VG: very good.

**Table 3:** Biochemical and Metabolic Activities of LAB and CoNS Isolates.

Isolates	Total Number	DL Lactic Acid	Acetoin Formation	Proteolytic Activity	Lipolytic Activity	Nitrate Reductase Activity	Decarboxylase Activity	Biofilm Formation
L1	60	57	47	7	0	6	1	NT
L2	18	18	18	0	0	2	0	NT
L3	13	11	12	1	0	1	0	NT
P1	3	3	3	0	0	0	0	NT
P2	1	1	1	0	0	0	0	NT
S1	24	NT	22	10	0	19	NT	0
S2	10	NT	10	0	0	10	NT	0
S3	6	NT	4	0	0	6	NT	1
S4	3	NT	3	0	0	3	NT	0
S5	2	NT	2	0	0	2	NT	0
S6	2	NT	2	0	0	1	NT	0
S7	1	NT	1	0	0	1	NT	0
S8	1	NT	1	0	0	1	NT	0
S9	1	NT	1	0	0	1	NT	0

L1: *L. plantarum*; L2: *L. paraplantarum*; L3: *L. sakei*; P1: *P. acidilactici*; P2: *P. pentosceus*; S1: *S. xylosus*; S2: *S. saprophyticus*; S3: *S. equorum*; S4: *S. simulans*; S5: *S. succinus*; S6: *S. carnosus*; S7: *S. hominis*; S8: *S. caprea*; S9: *S. vitulinus*, NT: Not tested.

**Table 4:** Antogonistic activity results of isolates.

Isolates	Number	*L. monocytogenes* ATCC 7644				*S. aureus* ATCC 25923				*S. aureus* ATCC 29213			
		Agar spot											
		N	P	G	VG	N	P	G	VG	N	P	G	VG
*L. plantarum*	60	-	-	53	7	15	3	42	-	15	1	44	-
*L. paraplantarum*	18	-	-	18	-	-	1	17	-	-	1	17	-
*L. sakei*	13	-	-	-	13	5	6	-	2	13	-	-	-
*P. acidilactici*	3	-	-	3	-	-	-	3	-	-	-	3	-
*P. pentosaceus*	1	-	-	1	-	-	-	1	-	-	-	1	-
		Well diffusion											
		N	P	G	VG	N	P	G	VG	N	P	G	VG
*L. plantarum*	60	59	-	1	-	60	-	-	-	60	-	-	-
*L. paraplantarum*	18	18	-	-	-	18	-	-	-	18	-	-	-
*L. sakei*	13	12	1	-	-	13	-	-	-	13	-	-	-
*P. acidilactici*	3	3	-	-	-	3	-	-	-	3	-	-	-
*P. pentosaceus*	1	1	-	-	-	1	-	-	-	1	-	-	-

N: negative, P: poor, G: good, VG: very good.

**Table 5:** Susceptibility of LAB and CoNS isolates to different antibiotics.

Isolates		Antibiotic Susceptibility									
	Number of Isolates	A	Cl	E	G	K	T	V	S	C	P
*L. plantarum*	60	55	60	58	-	-	54	1	1	52	54
*L. paraplantarum*	18	11	18	18	-	-	13	-	-	8	8
*L. sakei*	13	13	13	13	-	1	13	-	-	13	13
*P. acidilactici*	3	2	3	3	-	-	1	-	-	-	2
*P. pentosaceus*	1	1	1	1	-	-	1	-	-	-	-
*S. xylosus*	24	24	24	24	22	22	24	24	19	24	24
*S. saprophyticus*	10	10	10	10	10	10	10	10	9	10	10
*S. equorum*	6	6	6	6	6	6	6	6	6	6	6
*S. simulans*	3	3	3	3	3	3	3	3	3	3	3
*S. succinus*	2	2	2	2	2	2	2	2	2	2	2
*S. carnosus*	2	2	1	2	2	2	2	2	1	2	2
*S. hominis*	1	1	1	1	1	1	1	1	1	1	1
*S. caprea*	1	1	1	1	1	1	1	1	1	1	1
*S. vitulinus*	1	1	1	1	1	1	1	1	1	1	1

A: Ampicillin (10 µg), Cl: clindamycin (2 µg), E: erytromycin(15 µg), G: gentamicin (10 µg), K: kanamycin (30 µg), T: tetracycline (30 µg), V: vancomycin (30 µg), S: streptomycin (10 µg), C: cephalotin (30 µg), P: Penicillin G (10 U).

## Data Availability

The original contributions presented in this study are included in the article. Further inquiries can be directed to the corresponding author.
